# Rapid sequence evolution driven by transposable elements at a virulence locus in a fungal wheat pathogen

**DOI:** 10.1186/s12864-021-07691-2

**Published:** 2021-05-27

**Authors:** Nikhil Kumar Singh, Thomas Badet, Leen Abraham, Daniel Croll

**Affiliations:** Laboratory of Evolutionary Genetics, Institute of Biology, University of Neuchâtel, 2000 Neuchâtel, Switzerland

**Keywords:** Pathogen evolution, Crops, Genome-wide association mapping, Transposable elements, Genome assembly, Population genomics

## Abstract

**Background:**

Plant pathogens cause substantial crop losses in agriculture production and threaten food security. Plants evolved the ability to recognize virulence factors and pathogens have repeatedly escaped recognition due rapid evolutionary change at pathogen virulence loci (*i.e.* effector genes). The presence of transposable elements (TEs) in close physical proximity of effector genes can have important consequences for gene regulation and sequence evolution. Species-wide investigations of effector gene loci remain rare hindering our ability to predict pathogen evolvability.

**Results:**

Here, we performed genome-wide association studies (GWAS) on a highly polymorphic mapping population of 120 isolates of *Zymoseptoria tritici*, the most damaging pathogen of wheat in Europe. We identified a major locus underlying significant variation in reproductive success of the pathogen and damage caused on the wheat cultivar Claro. ﻿The most strongly associated locus is intergenic and flanked by genes encoding a predicted effector and a serine-type endopeptidase. The center of the locus contained a highly dynamic region consisting of multiple families of TEs. Based on a large global collection of assembled genomes, we show that the virulence locus has undergone substantial recent sequence evolution. Large insertion and deletion events generated length variation between the flanking genes by a factor of seven (5–35 kb). The locus showed also strong signatures of genomic defenses against TEs (*i.e.* RIP) contributing to the rapid diversification of the locus.

**Conclusions:**

﻿In conjunction, our work highlights the power of combining GWAS and population-scale genome analyses to investigate major effect loci in pathogens.

**Supplementary Information:**

The online version contains supplementary material available at 10.1186/s12864-021-07691-2.

## Background

Plant pathogens are a major threat to food security and cause annual losses of 20–30% of global harvest due to the lack of durable control strategies [1–3]. The emergence of new pathogens, the rise of new virulence in resident pathogens, or the gain in resistance against chemical control agents create significant challenges [2, 4, 5]. To design effective disease control strategies, understanding the molecular interaction between plants and pathogens is critical. The virulence of plant pathogens is largely determined by their repertoire of secreted proteins known as effectors [6, 7]. Effectors target a variety of different plant proteins and metabolic pathways to manipulate the immune response and physiological state of the host [8]. Plants evolved a large array of receptors often organized in networks that can directly or indirectly recognize the presence of effectors [7, 9, 10]. Detection of effectors triggers a variety of defense responses preventing the spread of pathogens across plant tissues. The discovery of resistance genes encoding receptors has provided key tools for the rapid breeding of resistant crop varieties [11, 12]. The identification of effectors in plant pathogens is challenging due to the large number of genes encoding effector-like proteins. The size of effector gene repertoires varies between filamentous pathogens [7, 13]. The potato light blight pathogen *Phytophthora infestans* has 1249 predicted effector candidates, whereas the white rust pathogen of *Arabidopsis thaliana, Albugo laibachii*, has only 143 predicted effector candidates [14]. The frequent birth and death of genes encoding effectors is underpinning at least part of the variation in candidate effector repertoires among species and underlies also variation within the same species [15]. Identifying functional effectors providing an advantage for a pathogen on a specific host remains challenging [8].

Effector gene polymorphism can be a major factor driving host-pathogen interactions [12, 15]. The analyses of complete fungal genomes in combination with mapping analyses significantly expanded our knowledge of effectors across major filamentous pathogens. Genome-wide association study (GWAS) and analyses of progeny populations revealed three effectors of the fungal wheat pathogen *Zymoseptoria tritici* [16–21]. The analyses of multiple completely assembled genomes revealed effector genes missing among individual isolates of the species [22–24]. Hence, pangenome analyses are crucial to establish the full extent of effector candidates within species [25]. Such effector polymorphism is thought to be at the origin of rapid gains in virulence [15, 26–28]. Breakdown in host resistance can be observed within few years following the deployment of a crop cultivar [29–32]. Effector gene evolution can be driven by the complete deletion of coding sequence, as well as the accumulation of point and frameshift mutations [15, 16, 33, 34].

The rapid evolution of effector gene sequences is often driven by features of the chromosomal sequence in which the effector genes are embedded. Effector genes can be located on lineage-specific accessory chromosomes [35–37]. Such accessory chromosomes are enriched in repetitive sequences [35]. Effector genes located on core chromosomes are often located in the most repetitive regions of the chromosome [38, 39]. The proximity to repetitive regions, in particular transposable elements (TEs), increases the likelihood for sequence rearrangements to occur. The localization of effectors in highly repetitive sub-telomeric regions contributed to rapid virulence evolution of the rice pathogen *Magnaporthe oryzae* [40, 41]. The *AVR-Pita* effector gene has been shown to undergo multiple translocations in the genome contributing to the evolution of virulence on specific hosts [42]. The insertion of a Mg-SINE TE in the effector gene *AvrPi9* led to a loss-of-function mutation enabling *M. oryzae* to escape host resistance [43]. The transposition of TEs can disrupt coding sequences or change the regulation of effector genes [19, 44, 45]. Additionally, ﻿repetitive sequences can lead to higher mutation rates through a mechanism known as repeat induced point (RIP) mutation [46–48]. *Brassica napus* (canola) carrying the *Rlm1* resistance gene suffered a breakdown of resistance against the fungal pathogen *L. maculans* [49]. The breakdown was associated with a rise in virulence alleles at the *AvrLm1* locus [49]. Sequence analyses revealed that the gain in virulence was driven by RIP mutations rendering the locus non-functional. Highly similar sequences nearby effector genes can also trigger ectopic recombination and, by this, the deletion or duplication of the effector gene. Consequently, the genomic context of effector genes provides critical information about effector evolvability. Hence, within-species analyses of effector gene diversification and TE dynamics of the surrounding regions have become key tools to retrace the evolution of virulence.

The haploid ascomycete *Zymoseptoria tritici* is one of the most destructive pathogens of wheat leading to yield losses of ~ 5–30% depending on climatic conditions [50, 51]. Pathogen populations across the wheat-producing areas of the world harbor significant variation in pathogenicity and genetic diversity [16, 17, 52–54]. GWAS were successfully used to identify the genetic basis of virulence on two distinct wheat cultivars [16, 17]. In addition, analyses of progeny populations revealed a third effector gene related to a resistance breakdown [19, 20]. GWAS was also successfully used to map the genetic architecture of a broad range of phenotypic traits related to abiotic stress tolerance [55]. TE dynamics are playing a key role in influencing the sequence dynamics at effector gene loci [16, 19, 44]. Gene gain and loss dynamics are accelerated in proximity to TEs [52]. TEs shape also the epigenetic landscape in proximity to effectors [44, 56]. Phenotypic traits expressed across the life cycle of the pathogen show extensive trade-offs possibly constraining the evolution of virulence [55, 57]. Identifying additional pathogenicity loci associated with host specificity remains a priority since for most wheat resistance genes (*i.e.* *Stb*), the corresponding effector genes remain unknown [58].

In this study, we aimed to identify the genetic basis of virulence on the wheat cultivar Claro using GWAS performed on a genetically highly diverse mapping population established from a single wheat field. We analyzed the expression patterns of genes in proximity to the top associated SNP, the presence of TEs and genetic variation at the locus in populations across the world to build a comprehensive picture of sequence dynamics at the newly identified virulence locus.

## Results

### Genome sequencing of a highly polymorphic pathogen field population

**Fig. 1:**
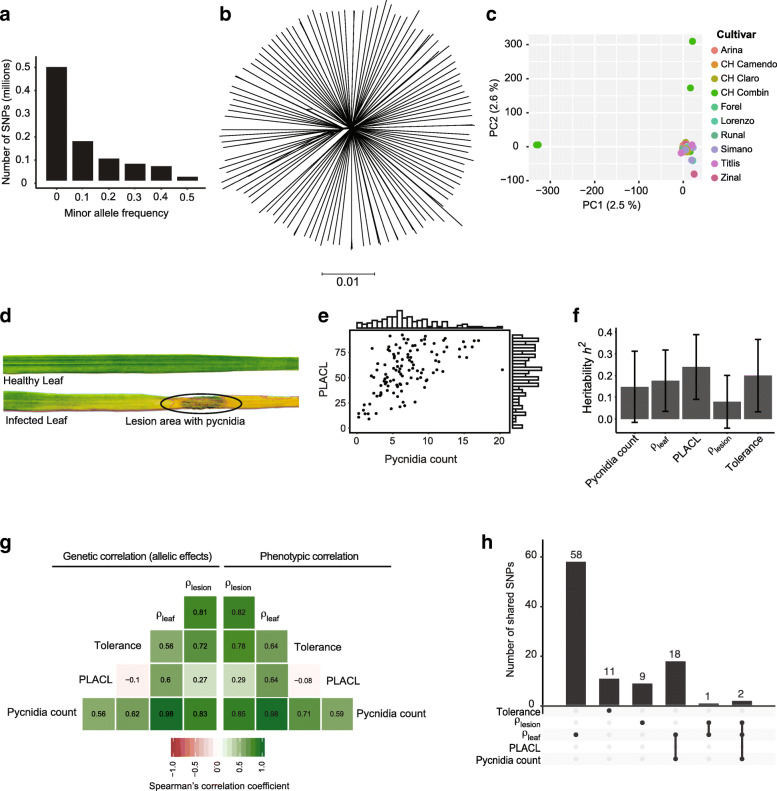
Genetic and phenotypic diversity in a single field population of Zymoseptoria tritici. **a** Minor allele frequency spectrum (frequency of the less common allele in the population) at 1′496’037 single nucleotide polymorphism (SNP) loci genotyped in 120 isolates. **b** Phylogenetic network of 120 isolates constructed using SplitsTree visualizing reticulation due to potential recombination. **c** The first two principal components (PC) from a PC analysis of 788′313 genome-wide SNPs with a minor allele frequency of at least 5%. Isolates are color-coded by the cultivar of the origin. **d** Photographs showing the difference between a mock treated and infected leaf. **e** Trait distribution of pycnidia counts in lesions and the percentage of leaf area covered by lesion (PLACL). **f** SNP based heritability (*h*_*2*_ SNP) of the virulence phenotypes estimated following a GREML approach. Error bars indicate standard errors. **g** Mean allelic effect (*i.e.*. genetic) correlation and phenotypic correlation coefficients for all measured virulence phenotypes. **h** Number of significantly associated SNPs (5% FDR threshold) exclusive to an individual virulence trait or shared among traits

To build a mapping population for GWAS, we used a subset of a previously established collection of 177 isolates of *Z. tritici.* The collection originates from a multi-year experimental wheat field in Switzerland planted with 335 wheat cultivars [54, 59] (Supplementary Table S1). In total 120 isolates from ten genetically different winter wheats (7–20 isolates per cultivar) collected at two different time points during a single growing season were included in this study. We analyzed whole-genome sequencing datasets of each isolate constituting an average coverage of 21X as previously described [54]. We found that the minor allele frequency (MAF) spectrum showed a strong skew towards rare alleles in the population, suggesting that the population did not experience any recent genetic bottlenecks (Fig. 1a). After filtering for MAF > 0.05 (also see methods), we obtained 788′313 high-confidence SNPs. We constructed an unrooted phylogenetic network using SplitsTree to visualize the genotypic differentiation within the population (Fig. 1b). Compared to the broader field population analyzed previously, our GWAS mapping population contained 10 clonal groups comprising a total of 21 isolates [54] (Supplementary Table S2). A principal component analysis confirmed the overall genetic differentiation within the population (Fig. 1c). Nearly all isolates were at similar genetic distances to each other with the exception of six isolates with larger genetic distances to the main cluster of isolates [54] (Fig. 1c). The percent variance explained was only 2.6 and 2.5 for principal component 1 and 2, respectively, though (Fig. 1c). Interestingly, the six isolates were all collected from cultivar CH Combin, which is susceptible to *Z. tritici* [60] and grouped into two clone groups of three isolates each (Supplementary Table S2). A principal component analysis performed after removing the six isolates collected from CH Combin revealed no meaningful population structure (Supplementary Fig. 1).

### Heritability and correlations among pathogenicity traits

We experimentally assessed the expression of pathogenicity traits of each individual isolate on the winter cultivar Claro using a greenhouse assay. The cultivar Claro was among the cultivars used in the multi-year experimental wheat field from which the isolates were sampled from [59]. The cultivar is widely planted in Switzerland and is generally mildly susceptible to *Z. tritici* [60]. We obtained quantitative data on symptom development from a total of 1′800 inoculated leaves using automated image analysis [59]. The image analyses pipeline was previously optimized to detect symptoms caused by *Z. tritici* under greenhouse conditions and uses a series of contrast analyses to obtain estimates of the surface covered by symptoms. For each leaf, we recorded the counts of pycnidia (structures containing asexual spores) and the percentage of leaf area covered by lesion (PLACL) (Fig. 1d-e). We considered the pycnidia count as a proxy for reproductive success of the pathogen on the host and PLACL as an indication of host damage due to pathogen infection. From these measurements, we derived three quantitative resistance measures: ρ_leaf_ is the pycnidia count per cm^2^ of leaf area, ρ_lesion_ is defined as the total number of pycnidia divided by per cm^2^ lesion area, and tolerance is expressed as the pycnidia count divided by PLACL. The overall reproductive success per leaf area is represented by ρ_leaf_ while ρ_lesion_ focuses on the reproductive success within the lesion area. Tolerance indicates the ability of the host to tolerate pathogen reproduction while limiting damage by lesions [61]. We found that the mean pycnidia count ranged from 0 to 20 (mean 7, median 6.3) among isolates and PLACL ranged from 2 to 97% (mean 56%, median 57.7%) (Fig. 1e, Supplementary Table S1, Supplementary Fig. 2). The values for ρ_leaf_ ranged from 0.04–7.2 (mean 2.4, median 2.15); ρ_lesion_ ranged from 0 to 13.8 (mean 3.6, median 3.3) and tolerance ranged from 0.15–0.3 (mean 0.12, median 0.17) (Supplementary Table S1, Supplementary Fig. 2).

We estimated SNP-based heritability (*h*^*2*^_*snp*_) for each trait using a genomic-relatedness-based restricted maximum-likelihood approach to partition the observed phenotypic variation (Fig. 1f). The *h*^*2*^_snp_ ranged from 0.08–0.23 among different phenotypes (Fig. 1f). Heritability for pycnidia counts and PLACL was 0.17 (SE = 0.14) and 0.15 (SE = 0.16), respectively. We found the highest *h*^*2*^_snp_ for ρ_leaf_ (0.24, SE = 0.15) exceeding *h*^*2*^_snp_ for ρ_lesion_ (0.19, SE = 0.16). Pathogenicity-related traits have overlapping genetic architectures leading to phenotypic and genetic correlations [55]. To identify potential trade-offs among traits, we analyzed correlations among all pairs of traits (Fig. 1g). We found overall positive phenotypic trait correlations except for PLACL and tolerance (*r*_*p*_ = − 0.08; Fig. 1g). To assess genetic correlations among traits, we performed GWAS on each trait. To avoid *p*-value inflation due to non-random degrees of relatedness among isolates, we used a mixed linear model that included a kinship matrix. We assessed the allelic effects across all SNPs for all traits to estimate the degree of genetic correlation among trait pairs. We found the genetic correlations (*r*_*p*_) to vary from − 0.1 to 0.98 (Fig. 1g). Pycnidia counts and ρ_leaf_ showed the highest degree of genetic correlation. Tolerance and PLACL showed the lowest degree of genetic correlation. Overall, phenotypic and genetic correlations among pairs of traits were highly similar.

### Major effect locus for pathogen reproduction on the cultivar Claro

**Fig. 2:**
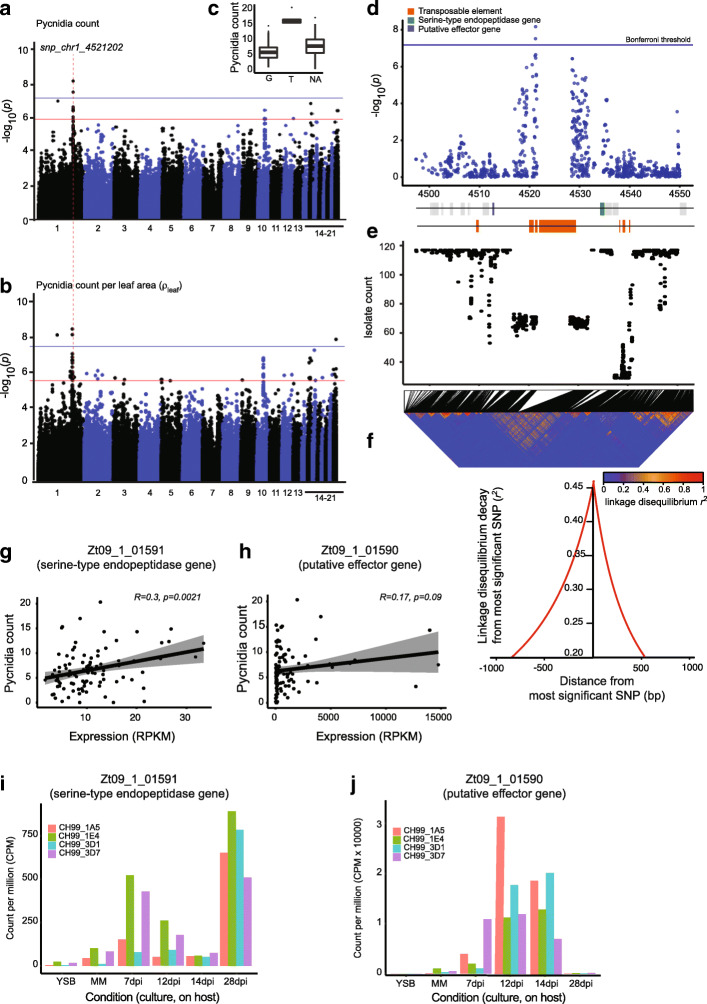
Genome-wide association mapping for virulence. Manhattan plots showing SNP marker association *p*-values for (**a**) pycnidia count and (**b**) ρleaf (pycnidia count per cm2 of leaf area). The genome-wide association mapping analyses was performed based on a mixed linear model including a kinship matrix. The blue and red lines indicate the significance thresholds for Bonferroni (⍺ = 0.05) and false discovery rate (FDR) at 5%, respectively. The dotted line represents the most significant association on chromosome 1 (snp_chr1_4521202). **c** Boxplot showing the pycnidia counts of isolates carrying the reference allele G or alternative allele T at the top significant SNP. **d** Zoomed in Manhattan plot for association *p*-values of SNPs in a ~ 25 kb region centered on the top SNP snp_chr1_4521202. Horizontal lines represent the Bonferroni threshold (⍺ = 0.05). **e** Genotyping rates of SNPs in the mapping population. **f** Linkage disequilibrium *r*^2^ heatmap of the entire region. Linkage disequilibrium decay plot focused on the most significantly associated SNP with nearby SNPs. **g-h** Correlation plot of pycnidia count with gene expression of the flanking effector candidate gene (Zt09_1_01590) and the serine-type endopeptidase gene (Zt09_1_01591). I-J) Transcriptional profiling of the effector gene and the serine-type endopeptidase gene on wheat 7, 12, 14, and 28 days post infection

We used the GWAS on each trait to identify the most significantly associated SNPs in the genome. We focused on association *p*-values passing the 5% false discovery rate threshold for all the phenotypes except for PLACL where we found no significant associations (Supplementary Fig. 3). All significantly associated SNPs for pycnidia count were overlapping with significantly associated SNPs for ρ_leaf_ and ρ_lesion_ (Fig. 1h). The traits ρ_leaf,_ ρ_lesion_ and tolerance had 58, 9 and 11 associated SNPs, respectively, which were uniquely associated with the specific trait and not overlapping with any other trait (Fig. 1h). We then focused our investigation on the most significantly associated SNPs passing the Bonferroni threshold (⍺ = 0.05). We found a single locus on chromosome 1 with significantly associated SNPs for pycnidia count, ρ_leaf_ and ρ_lesion_ (Fig. 2a-b, Supplementary Fig. 3C, E). Both integrating principal components of a principal component analysis (PCA) and a kinship matrix can be used as random factors to control false positive rates in a GWAS. The inclusion of principal components did not meaningfully affect the outcome and confirmed the single strong association on chromosome 1 for pycnidia count, ρ_leaf_ and ρ_lesion_ (Supplementary Fig. 4B,D-E). The top SNP (chr1_4521202) showed an association of isolates carrying the non-reference allele T with higher pycnidia production compared isolates with reference allele G (Fig. 2c). The non-reference allele was less frequent in the population (10%) and nearly half (48%) of all isolates were not assigned a SNP genotype at the locus.

We analyzed sequence characteristics of the chromosomal region surrounding the top locus. The SNP *chr1_4521202* is located in an intergenic region rich in TEs (Fig. 2d-e). The closest identified genes include a gene encoding a putative effector (Zt09_1_01590) and a gene encoding a serine-type endopeptidase (Zt09_1_01591). The effector gene (415 bp) in length have four SNPs detected in the mapping population. Additionally, the gene encodes a protein of 114 amino acids with 7% cysteine residues and is predicted to be secreted. We detected no evidence for a conserved protein domain using PFAM. The two genes were at a distance of ~ 8 kb and ~ 4.5 kb, respectively, from the SNP *chr1_4521202* (Supplementary Table S3). The low genotyping rate at the SNP suggests that segmental deletions are present. The genotyping rate was 58%, which is consistent with the SNP genotyping rate for nearby SNPs (within ~ 5 kb; Fig. 2e). We recovered no SNPs in the immediate vicinity (at around 4.25 Mb on chromosome 4). The genotyping rate increases to close to 100% at a further distance of the top SNP (> 10 kb; Fig. 2e. The segmental pattern in the reduced genotyping rate close to the most significant SNP suggests that a substantial fraction of the isolates harbor deletions. We analyzed patterns of linkage disequilibrium among pairs of SNPs including SNP *chr1_4521202* (Fig. 2f). We found that the decay in linkage disequilibrium generally occurred at short distance near the associated virulence locus. The linkage disequilibrium in the effector gene region decayed to *r*^2^ = 0.2 within ~ 1000 bp while the decay in the repeat rich region surrounding the most significantly associated SNP was faster (*r*^*2*^ = 0.2 within ~ 500 bp; Fig. 2f). The increased linkage disequilibrium suggests that the physical distance among SNPs in the analyzed isolates is shorter consistent with the detection of deletions.

We analyzed transcription levels of the two closest genes using RNA-seq data generated under culture conditions simulating starvation (minimal medium) for all isolates of the GWAS panel. Both genes were conserved in all the isolates and appear transcriptionally active with variable expression levels among the isolates. The candidate effector gene was transcribed between 12 and 14′750 reads per kilobase of transcript per million mapped reads (RPKM) (Fig. 2h, Supplementary Fig. 5). RPKM normalization compensates for library size differences and for the bias generated by the higher number of reads from longer RNA molecule [62]. The serine-type endopeptidase gene showed much lower transcription ranging from 1.6–33.4 RPKM (Fig. 2g, Supplementary Fig. 5). We found that transcription levels of the gene encoding the endopeptidase was positively correlated with the amount of pycnidia produced (*r* = 0.3, *p* = 0.0021, Fig. 2g). We found no significant correlation between pycnidia production and expression of the effector candidate gene (Fig. 2h). We also investigated transcriptional activity of the genes during wheat infection. For this, we analyzed RNA-seq data of four isolates previously collected from a nearby site in Switzerland and for which *in planta* transcriptional profiles were available [44, 63]. The effector gene *Zt09_1_01590* is upregulated during early infection stages (7–14 days post infection) while the endopeptidases gene *Zt09_1_01591* is mainly expressed towards the end of the infection cycle (~ 28 days post infection; Fig. 2i-j).

### Transposable element dynamics and sequence rearrangements

**Fig. 3:**
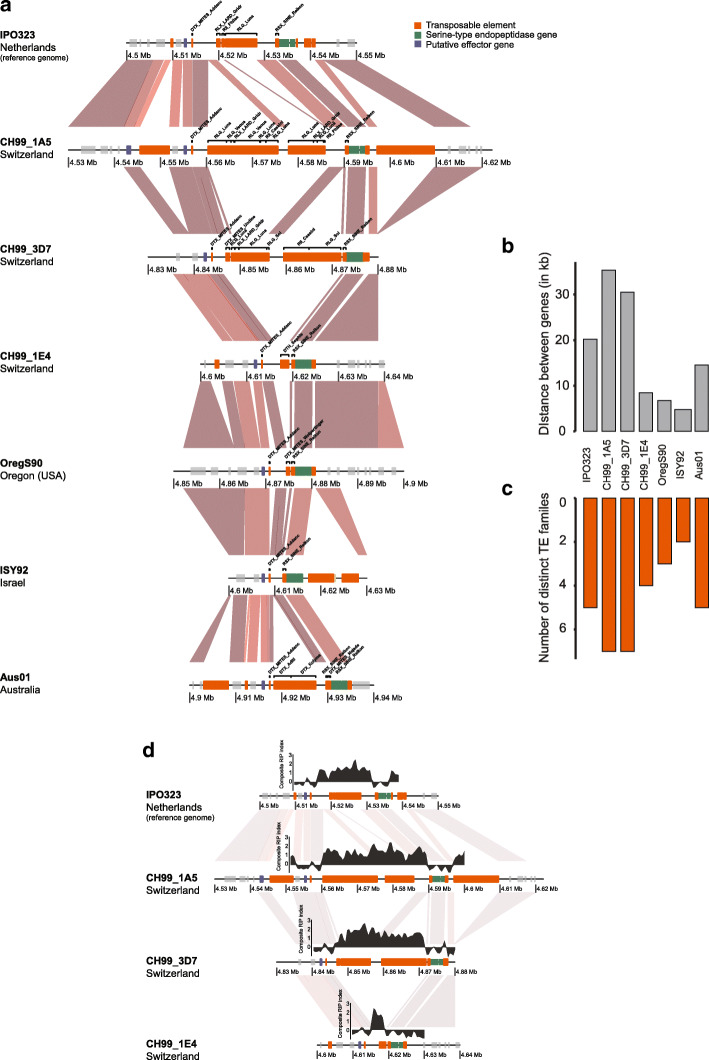
TE content variation at the virulence locus. **a** Synteny plot of the top locus analyzed in seven completely assembled genomes. The red gradient segments represent the percentage of sequence identity from BLASTN alignments. Darker colors indicate higher identity. **b** Distance variation between the two genes surrounding the top locus (Zt09_1_01590 and Zt09_1_01591). **c** The number of different TE families found at least once per isolate at the top locus. **d** Repeat induced point (RIP) mutation signatures in the topic locus. The Large RIP Affected Regions (LRARs) composite index was calculated using the RIPper tool (van Wyk et al., 2019)

Given the indications for segmental deletions at the virulence locus, we analyzed multiple completely assembled genomes of the species. We included genomes from isolates from Switzerland, United States, Australia and Israel covering the global distribution range of the pathogen [24]. The locus showed a highly variable content in TEs underlying significant length variation. The distance between the two flanking genes is 20.2 kb in the reference genome IPO323 used for mapping (Fig. 3a-b). However, this distance varies from 4.8–35.3 kb between the genes depending on the genome for an average distance of ~ 17 kb (Fig. 3b). The longest distance between genes was found in the genome of the Swiss strain CH99_1A5 and the shortest distance was found in the genome of the Israeli strain ISY92.

We identified five different TE families in the reference genome IPO323 covering a segment of ~ 20 kb (Fig. 3c). We detected additional TE families in two of the three genomes from Switzerland (CH99_1A5 and CH99_3D7). The genomes carry multiple copies of a total of seven different TE families. Meanwhile, the two genomes from Israel and the United States showed a reduction in TEs with the region carrying only single copies of two and three different TE families, respectively (Fig. 3a-c). The presence of TEs in fungal genomes can trigger RIP mutations. We found consistent signatures of RIP between the two flanking genes but we found no indications for RIP leakage into the flanking genes (Fig. 3d, Supplementary Fig. 6).

### Transposable element insertion dynamics across populations

**Fig. 4:**
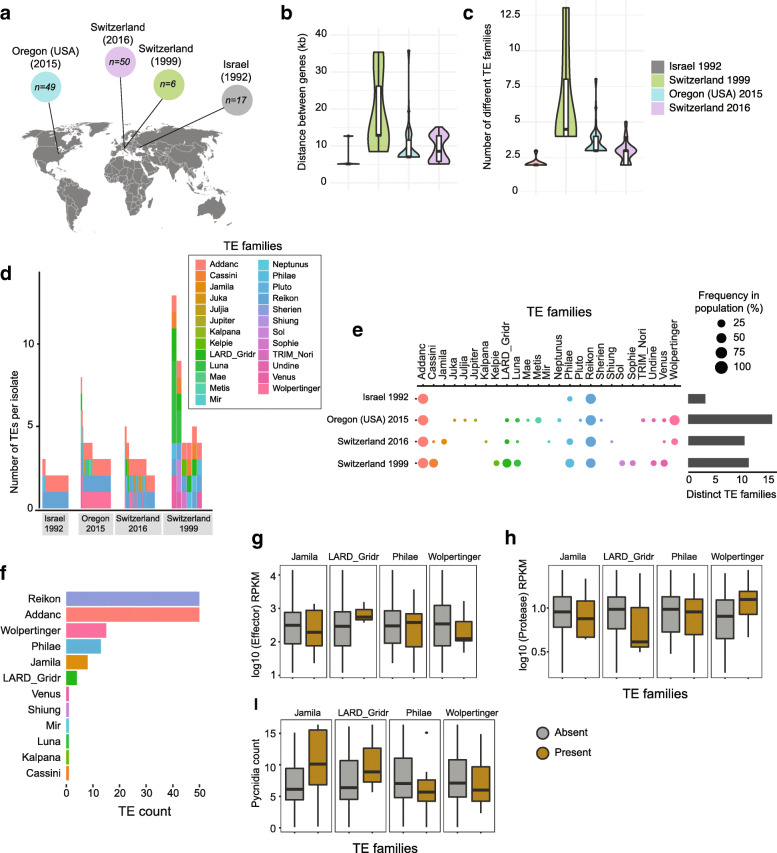
Analysis of transposable element dynamics across continents. **a** Analysis of 122 isolates for which a draft genome assembly produced a scaffold containing both flanking genes Zt09_1_01590 and Zt09_1_01591. **b** Boxplot showing variation in the distance between the two genes per population. **c** TE content variation of the sequence flanked by the two genes. **d** Total TE copies in the sequence flanked by the two genes. **e** Frequency of the TE families in the sequence flanked by the two genes as a percentage of the population. **f** Frequency of TE families among the isolates from the GWAS mapping population (*n* = 50 with a scaffold spanning both genes) (**g-i**) Boxplots showing the expression of the genes Zt09_1_01590 and Zt09_1_01591, and pycnidia counts, respectively, for isolates carrying or not specific TEs at the top locus

The small set of completely assembled genomes provides only a partial view on the sequence rearrangement dynamics within the species. Hence, we generated draft genome assemblies for 432 isolates from previously analyzed field populations in the United States (*n* = 56 + 97), Switzerland (*n* = 37 + 185), Israel (*n* = 30) and Australia (*n* = 27; Supplementary Table S4). The two population from United States and Switzerland were collected at an interval of 25 and 20 years, respectively, from the same field (Supplementary Table S4). Illumina sequencing datasets for fungi with compact genomes produce reasonably accurate draft assemblies [64–66]. We used BLASTN [67] to locate the two genes *Zt09_1_01590* and *Zt09_1_01591* adjacent to the top SNP across all assemblies. We retained only draft assemblies for which both genes were located on the same scaffold. Hence, these scaffolds provide a contiguous view on the sequences located between the two adjacent genes. With this filtering step, we retained 122 isolates from all four different locations including the United States (*n* = 49), Israel (*n* = 17) and Switzerland (*n* = 6 and 50) (Fig. 4a; Supplementary Table S4). The distance between the two genes ranged from 5 to 35 kb, which is highly consistent with the gene distances observed in the completely assembled genomes (Fig. 4b). The isolates from Israel and Switzerland (collection 2016) showed shorter distance ranging from 5 to 15 kb. The United States population and the older Switzerland population (collection 1999) showed a range of 6.8–35 kb between the genes (Fig. 4b).

We annotated the scaffolds matching the top GWAS locus using consensus sequences of known TE families. Overall, the TEs between the two adjacent genes grouped into 11 superfamilies and 25 families (Fig. 4d). The two most frequent TEs included both retrotransposons and miniature-inverted repeat transposable elements. We found that genomes from the United States and the earlier Switzerland population (collection 1999) had higher TE copy numbers compared to other genomes from the other populations (2–13 TE copies; Fig. 4c). The locus contains overall 16 different TE families in the United States population (Fig. 4d-e). The locus contained 3, 11 and 16 different TE families in the Israeli, and the Swiss 1999 and 2016 populations, respectively (Fig. 4d-e). The TEs RSX_SINE_Reikon and DTX_MITES_Addanc were found in all analyzed populations population while other TE families were segregating in populations in different proportions (Fig. 4e). To test for potential associations of TE presence and pathogenicity traits, we focused on the complete scaffolds retrieved from 50 different isolates of the GWAS population (Fig. 4f). We found segregating presence-absence polymorphism for the four TE families RII_Philae and RLX_LARD_Gridr (retrotransposons), as well as DTX_MITES_Wolpertinger and DTC_Jamila (DNA transposons; Fig. 4f). None of the TE presence-absence polymorphism showed a significant association with the transcription of adjacent genes (Fig. 4g-h; effector candidate *Zt09_1_01590*: Student’s *t*-test, *p* > 0.15; serine-type endopeptidase *Zt09_1_01591*: Student’s *t*-test, *p* > 0.05). We also found no significant association with the TE presence-absence polymorphism and reproduction on the host (Fig. 4i; Student’s *t*-test, *p* > 0.2).

## Discussion

We used whole-genome sequencing data and association mapping to unravel the genetic architecture of pathogenicity of *Z. tritici* on the wheat cultivar Claro. The identified locus is rich in TEs and is flanked by genes encoding an effector candidate and a serine-type endopeptidase. We analyzed a worldwide set of populations to analyze sequence variation at the pathogenicity locus. We found significant length variation caused by the insertion of a diverse set of TEs.

Variation in pathogenicity on the wheat cultivar Claro was largely quantitative. We found that heritability was higher for pathogen virulence (damage to host) than pathogen reproduction (production of pycnidia). This is in contrast to analyses of heritability across 12 different wheat cultivars where heritability for pathogen reproduction was typically higher compared to lesion damage [55]. However, virulence and reproduction were overall positively correlated in both studies. We also found a high degree of phenotypic and genetic correlation with tolerance (*i.e.* preventing lesion damage despite high reproduction of the pathogen). Using GWAS, we identified several loci significantly associated with different pathogenicity traits. The most significant associations were found for pycnidia counts and ρ_leaf_ both related to reproductive success of the pathogen. Pathogen reproduction showed a strong single locus association while host damage (*i.e.* lesions) revealed no single gene effects. The difference between traits may be due to the fact that the genetics underlying host damage is more complex. Lesions are caused by host cell death triggered as a response to pathogen attack [68, 69]. Hence, variation in lesion development among isolates could be due to the host’s ability to perceive specific molecules produced only by a subset of the isolates [12, 22, 70]. Furthermore, variation in the pathogen’s ability to spread across tissue and manipulate host immune responses could also lead to variation in overall lesion development [71]. Interestingly, extensive lesion development is not necessarily related to pycnidia production by the pathogen across cultivars [61, 72]. This suggests that despite damage to the leaf, the host immune system can efficiently repress the pathogen from acquiring nutrients to reproduce. In contrast, the strong single locus association for pycnidia production on the cultivar Claro suggests a rather simple genetic architecture. Hence, the action of a single pathogen factor (e.g. an effector) may be largely sufficient to determine variation in host exploitation and reproduction.

We identified a highly polymorphic chromosomal locus associated with pathogenicity on the cultivar Claro. The most significant SNPs mapped in an intergenic region flanked by a large cluster of diverse TEs. We found no clear evidence for a coding sequence in immediate proximity of the most significantly associated SNPs. The closest genes encode functions, which may be relevant for host infection though. Serine-type endopeptidases play pivotal roles in nutrient degradation and subsequent assimilation, as well as protection from the host immune system [73]. Serine proteases can also help the pathogen to escape the host’s immune system by degrading chitinases targeted at the fungal cell wall [74]. Furthermore, serine proteases play a role in the nutrient acquisition from plant tissue [75] and potentially during the initiation of necrosis [76]. The second gene encodes a putative effector, which is a category of genes showing frequent presence-absence polymorphism within the species [25, 52]. Our analyses of linkage disequilibrium decay suggest that neither of the two adjacent genes play a causal role in pathogenicity on Claro. The most dramatic changes occurred due to the insertion and deletion of TEs next to the most significantly associated SNPs. The TE dynamics diversified the locus to the extent that the distance between the adjacent genes varies by a factor of seven (5–35 kb). The insertion and deletion of TEs can have both an impact on gene regulation by inducing epigenetic silencing or upregulation. Both mechanisms are well established in *Z. tritici* and underlie variation in melanin production, virulence and fungicide resistance [19, 77, 78]. The locus flanked by the two genes showed strong signatures of RIP. The elevated mutation rates triggered by this genomic defense mechanism against TEs likely contributed to the rapid diversification of the locus. Yet, we could not establish any direct association between the insertion of individual TEs and the expression of pathogenicity. Targeted deletion assays focusing on individual sequence segments may provide experimental evidence for the sequence variation underlying pathogenicity on the wheat cultivar.

## Conclusions

The effects of gene-TE proximity have been studied mainly in animal [79, 80] or plant models [81]. Only a handful studies are focused on fungi. Some fungal pathogens have genomes with a clearly compartmentalized architecture described by the two-speed model [26]. The core genome encodes all essential genes while niche- or host-specific genes (e.g. effectors) are typically encoded in the repeat-rich genome compartment. Such genome architectures have been identified in *Mycosphaerella fijiensis* [82], *Cochliobolus heterostrophus* [83]*, Fusarium* species [84]*, L. maculans* [34] and *Verticillium* species [85]. However, systematic investigation of TEs and co-localizing genes have rarely been extended to the within species level. Our study shows that a combination of genome-wide association mapping, complete and draft genome assemblies can provide a comprehensive insight into the evolutionary dynamics of virulence loci. Hence, even in absence of experimentally validated effectors, the evolutionary trajectory of virulence loci becomes tractable. Our approach should be broadly applicable to many fungal pathogen systems.

## Methods

### Field collection and storage

*Z. tritici* isolates were collected from the Field Phenotyping Platform (FIP) site of the ETH Zürich, Switzerland (Eschikon, coordinates 47.449°N, 8.682°E). We analyzed a total of 120 isolates collected during the 2015/2016 growing season from 10 winter wheat cultivars, which are commonly grown in Switzerland [86]. We analyzed isolates originating from two collection time points over the season (Table S1). Isolates from the first collection (*n* = 62) were collected when wheat plants were in Growth stage (GS) 41 while the second collection (*n* = 58) was performed when the plants were in GS 85 stage. After sampling, spores of each isolate were stored in either 50% glycerol or anhydrous silica gel at − 80 °C. Additional information regarding sampling schemes and genetic diversity is available [54].

### Culture preparation and seedling infection assay

Isolates were revived from glycerol stock by adding 50 μl fungal stock solution to a 50 ml conical flask containing 35 ml liquid YSB (yeast-sucrose broth) medium. The inoculated flasks were incubated in the dark at 18^°^C and 140–180 rpm on a shaker-incubator. After 8 days of incubation, the cultures were passed through four layers of meshed cheesecloth and washed twice with sterile water to remove media traces. The filtering step also largely eliminated hyphal biomass but retained spores. The Swiss winter wheat cultivar Claro was used for virulence assays (provided by DSP Delley, Inc.). Four seeds were sown in pots with commercial compost soil in triplicates. The pots were frequently in the growth chamber. The plants were grown under controlled conditions as follows: 16/8 h day/night periods at 18 °C throughout the experiment. The growth chamber was maintained at 70% humidity. Plants were grown for 3 weeks before infection with *Z. tritici*. To initiate infections, washed spores were diluted to 2 × 10^5^ spores/ml in 15 ml of sterile water containing 0.1% TWEEN20. For each isolate, plants from three pots were infected using spray bottles. After spray inoculation, the plants were allowed to dry before sealing them in clear plastic bags to maintain 100% humidity for 48 h. Plastic bags were removed after 48 h and conditions were kept as described above.

### Automated image-based evaluation of infection

Twenty-one days post inoculation (dpi), the second leaf of each plant was cut and fixed on a barcoded white paper. Leaves were scanned immediately using a flatbed scanner at 1200 dpi. The scanned images were batch-processed using a macro [59, 87] based on routines implemented in the image analysis software ImageJ (Rasband, W.S., ImageJ; U. S. National Institutes of Health, http://imagej.nih.gov/ij/, 1997–2012). Briefly, the macro recorded the total leaf area, total lesion area, the number of pycnidia, mean size of pycnidia and pycnidia grey value. The percent leaf area covered by lesions (PLACL) was calculated as the ratio of the total lesion area and total leaf area [72].

### Whole-genome sequencing, variant calling and RNA-seq analyses

Approximately 100 mg of lyophilized spores were used to extract high-quality genomic DNA using the Qiagen DNeasy Plant Mini Kit according to the manufacturer’s protocol. We sequenced paired-end reads of 100 bp each with an insert size of ~ 550 bp on the Illumina HiSeq 4000 platform. Raw reads are available on the NCBI Sequence Read Archive under the BioProject PRJNA596434 [88]. For RNA sequencing, the same isolates were cultured in a Vogel Minimal N Medium [89] where ammonium nitrate was replaced with potassium nitrate and ammonium phosphate [90]. The medium contained no sucrose and agarose to induce hyphal growth. Total RNA was isolated from the filtered mycelium after 10–15 days using the NucleoSpin® RNA Plant and Fungi kit. The RNA concentration and integrity were checked using a Qubit 2.0 Fluorometer and an Agilent 4200 TapeStation System, respectively. Only high-quality RNA (RIN > 8) was used to prepare TruSeq stranded mRNA libraries with a 150 bp insert size and sequenced on an Illumina HiSeq 4000 in the single-end mode for 100 bp.

### Sequencing filtering and analysis

We performed sequencing quality checks using FastQC v. 0.11.9. (Andrews S., 2010) and extracted read counts. Sequencing reads were then trimmed for adapter sequences and sequencing quality using Trimmomatic v. 0.39 [91] using the following settings: illuminaclip = TruSeq3-PE.fa:2:30:10, leading = 10, trailing = 10, sliding-window = 5:10 and minlen = 50. Trimmed sequencing reads were aligned to the reference genome IPO323 [92]; accessible from https://fungi.ensembl.org/Zymoseptoria_tritici/Info/Index) and the mitochondrial sequence (European Nucleotide Archive EU090238.1) using Bowtie2 v. 2.4.1 [93]. Multi-sample joint variant calling was performed using the HaplotypeCaller and GenotypeGVCF tools of the GATK package v. 4.0.1.2 [94]. We retained only SNP variants (excluding indels) and proceeded to hard filtering using the GATK VariantFiltration tool based on the following cutoffs: QD < 5.0; QUAL < 1000.0; MQ < 20.0; − 2 > ReadPosRankSum > 2.0; − 2 > MQRankSum > 2.0; − 2 > BaseQRankSum > 2.0. Finally, we applied a filtering for a per SNP locus genotyping rate of at least 50% (“--max-missing” option) and a minor allele count (MAC) of 1 using VCFtools v. 0.1.15 [95]. We further subset SNPs for GWAS by requiring a minor allele frequency (MAF) of at least 0.05 (5%). Similarly, RNA-seq datasets were checked for quality using FastQC v. 0.11.9. and trimmed with Trimmomatic v0.39 to remove adapter sequences and low-quality reads with parameters: illuminaclip: TruSeq3-SE.fa:2:30:10 leading = 3, trailing = 3, sliding-window = 4:15 and minlen = 36. Trimmed sequences were aligned to the reference genome IPO323 using HISAT2 v. 2.1.0 [96] with the parameter “--RNA-strandedness reverse”.

### Population genetic analyses

Population structure and relatedness among individuals in the mapping population may be a source of *p*-value inflation due to non-random phenotype-genotype associations [97, 98]. To account for this, we analyzed the population structure and genetic relatedness of all isolates by performing a PCA. We performed and visualized the PCA using the R packages vcfR v. 1.8.0 [99], adegenet v. 2.1.1 [100], ade4 v. 1.7–13 [101] and ggplot2 v. 3.1.0 [102]. We also generated an unrooted phylogenetic network using SplitsTree v4.14.6 [103]. File format conversions were performed using PGDSpider v2.1.1.5 [104]. To identify groups of clonal isolates, we calculated the pairwise genetic distances between all isolates using the function “dist.dna” included in the R package ape v. 5.3 [105]. Isolate pairs with a pairwise genetic distance below 0.01 were considered as clones for further analyses (see [54] for more details). The SNP-based heritability (*h*^*2*^_snp_; equivalent to narrow-sense heritability) for each trait was estimated using the genome-wide complex trait analysis (GCTA) tool v.1.93.0 [106]. The *h*^*2*^_snp_ was estimated using a genome-based restricted maximum likelihood (GREML) approach using the phenotypic values of each trait and considering the additive effect of all the SNPs represented by the GRM.

### Genome-wide association mapping and linkage disequilibrium analyses

We performed GWAS based on mixed linear models accounting for genetic relatedness using either only a kinship matrix (MLM K) or a kinship matrix along with principal components of a PCA (MLM K + Q). We estimated relatedness among isolates by computing a kinship matrix using the scaled identity-by-state (IBS) algorithm implemented in TASSEL v. 20,201,110 [107]. We included the kinship matrix as a random effect in the mixed linear models for association mapping using TASSEL. We used the allelic effect output of TASSEL to compute the pairwise genetic correlation (Spearman’s correlation) values using complete observations (use = pairwise.complete.obs) and visualized the values using the *ggcorr* function from the GGally R package v2.1.1 [108]. Similarly, we used the Spearman’s correlation to compute pairwise phenotypic trait correlations. Association mapping outcomes were visualized using the R package *qqman* v 0.1.4 [109]. We considered associations to be significant when *p*-values were smaller than the Bonferroni threshold at the nominal α = 0.05 (here *p* < 1.1e-7). The Bonferroni threshold was calculated by dividing the nominal threshold of 0.05 by the total number of SNPs used for GWAS. False discovery rate (FDR) thresholds of 5% were determined using the *p.adjust* function in the stat package in R. We explored the genomic regions containing significantly associated loci using the “closest” command in bedtools v. 2.29.0 [110]. Regions in the genome spanning the most significant associations were investigated for linkage disequilibrium patterns. We calculated the linkage disequilibrium *r*^2^ between marker pairs using the “option–hap-r2” in VCFtools v. 0.1.15 [95] with “--ld-window-bp” of 10,000. A heatmap was generated based on the *r*^2^ values with the R package *LDheatmap* v 0.99–7 [111].

### De novo genome assemblies, TE annotation, synteny analyses

We analyzed the locus surrounding the genes *Zt09_1_01590* and *Zt09_1_01591* in multiple completely assembled genomes of isolates collected in Switzerland, the United States, Australia and Israel covering the global distribution range of the pathogen (Badet et al., 2020). For synteny plots, the available repeat-masked chromosome-scale assemblies were analyzed using pairwise BLASTN. Information on BLAST hits among homologous chromosomes was visualized in R using the *genoplotR* package [112]. We analyzed signatures of repeat induced point mutations (RIP) using The RIPper online tool available at https://theripper.hawk.rocks/ [113].

To analyze sequence polymorphism at the locus, we used draft genome assemblies of 432 isolates from previously analyzed field populations in the United States, Switzerland, Israel and Australia [16, 88]. Illumina short read data was obtained from the NCBI Sequence Read Archive under the BioProject PRJNA327615 [16] and PRJNA596434 [88]. We used SPAdes version 3.14.0 to produce draft assemblies for each isolate [114]. We ran the tool with the following settings: -k 21,33,55,75,95 --careful. De novo assemblies were annotated for TEs using the TE consensus sequences (https://github.com/crolllab/datasets) generated for the species [24]. Consensus sequences were previously manually curated and renamed based on the three-letter classification system [115, 116]. The curated consensus sequences were used for annotation of each individual de novo assembly using RepeatMasker version 4.0.8 with a cut-off value set to 250 [117], ignoring simple repeats and low complexity regions. Further filtering of the TE annotation included: (1) removal of element annotations shorter than 100 bp, (2) merging of identical adjacent TE families overlapping by more than 100 bp, (3) renaming of overlapping TE families overlapping by more than 100 bp as nested insertions, and (4) grouping of interrupted elements separated by less than 200 bp into a single element using a minimal distance between start and end positions.

## Supplementary Information

**Additional file 1: Supplementary Fig. 1:** The first two principal components (PC) of 788′313 genome-wide SNPs in 114/120 isolates. Isolates are color-coded by the cultivar of the origin. The six excluded isolates grouped into two clone groups of three isolates each and were collected from cultivar CH Combin. **Supplementary Fig. 2:** Phenotypic trait values. Red and blue lines represent the mean and median, respectively. A) The percentage of leaf area covered by lesion (PLACL). B) pycnidia count. C) Tolerance expressed as the pycnidia count divided by PLACL. D) ρ_leaf_ is the pycnidia count per cm^2^ of leaf area and E) ρ_lesion_ is defined as the total number of pycnidia divided by per cm^2^ lesion area. **Supplementary Fig. 3:** Manhattan and QQ-plot representing of the genome-wide association mapping analyses using mixed linear models based on a kinship matrix. The blue line corresponds to the Bonferroni threshold (alpha = 0.05) and the red line corresponds to the 5% FDR. A-B) Percentage of leaf area covered by lesions (PLACL), C-D) tolerance E-F) ρ_lesion_. **Supplementary Fig. 4:** Manhattan and QQ-plot representing of the genome-wide association mapping analyses. The GWAS was performed using mixed linear models including a kinship matrix and the first two principal components as random factors. The blue line corresponds to the Bonferroni threshold (alpha = 0.05) and the red line corresponds to the 5% FDR. A-B) Percentage of leaf area covered by lesions (PLACL), C-D) Pycnidia count, E-F) tolerance G-H) ρ_leaf_ and I-J) ρ_lesion_. **Supplementary Fig. 5:** Gene expression in reads per kilobase of transcript, per million mapped reads (RPKM) for genes closest to the top-associated SNP. A) Putative effector gene (*Zt09_1_01590*) and B) serine-type endopeptidase gene (Zt09_1_01591). **Supplementary Fig. 6:** The Large RIP Affected Regions (LRARs) composite index was calculated using The RIPer tool (van Wyk et al., 2019) and shown in black. The region flanked by the genes *Zt09_1_01590* and *Zt09_1_01591* is shown in complete genome assemblies of seven isolates from global population.

**Additional file 2 Supplementary Table S1:** Phenotypic trait values used for GWAS. The percentage of leaf area covered by lesion (PLACL); ρ_leaf_ is the pycnidia count per cm^2^ of leaf area; ρ_lesion_ is defined as the total number of pycnidia divided by per cm^2^ lesion area. Tolerance is expressed as the pycnidia count divided by PLACL. **Supplementary Table S2:** Groups of clonal genotypes identified in the GWAS population with information about the collection time point and cultivar of origin. The clonal genotype columns provides a unique identifier. See Singh et al. (2020) for more detailed analyses. **Supplementary Table S3:** List of significantly associated SNPs above 5% FDR for pycnidia counts. **Supplementary Table S4:** Number of isolates from populations on different continents analyzed for transposable element content. Total assembled genomes and total of isolates per population where a scaffold was retrieved containing both genes *Zt09_1_01590* and *Zt09_1_01591*.

## Data Availability

Illumina short reads were retrieved from the NCBI Sequence Read Archive (BioProject accessions PRJNA327615, PRJNA596434 and PRJNA650267) accessible from https://www.ncbi.nlm.nih.gov/sra. All other data are reported in Supplementary Information.

## References

[1] Chakraborty S, Newton AC (2011). Climate change, plant diseases and food security: an overview. Plant Pathol.

[2] Strange RN, Scott PR (2005). Plant disease: a threat to global food security. Annu Rev Phytopathol.

[3] Savary S (2020). Plant health and food security. J Plant Pathol.

[4] McCann HC (2020). Skirmish or war: the emergence of agricultural plant pathogens. Curr Opin Plant Biol.

[5] Subbarao KV, Sundin GW, Klosterman SJ (2015). Focus Issue Articles on Emerging and Re-Emerging Plant Diseases.

[6] Rovenich H, Boshoven JC, Thomma BP (2014). Filamentous pathogen effector functions: of pathogens, hosts and microbiomes. Curr Opin Plant Biol.

[7] Depotter JRL, Doehlemann G (2020). Target the core: durable plant resistance against filamentous plant pathogens through effector recognition. Pest Manag Sci.

[8] Selin C, de Kievit TR, Belmonte MF, Fernando WGD. Elucidating the role of effectors in plant-fungal interactions: Progress and challenges. Front Microbiol. 2016;7. 10.3389/FMICB.2016.00600. 10.3389/fmicb.2016.00600 PMC4846801 27199930

[9] van der Burgh AM, Joosten MHAJ (2019). Plant immunity: thinking outside and inside the box. Trends Plant Sci.

[10] Wu C-H, Abd-El-Haliem A, Bozkurt TO, Belhaj K, Terauchi R, Vossen JH (2017). NLR network mediates immunity to diverse plant pathogens. Proc Natl Acad Sci.

[11] Vleeshouwers VGAA, Oliver RP (2014). Effectors as tools in disease resistance breeding against biotrophic, Hemibiotrophic, and Necrotrophic plant pathogens. Mol Plant-Microbe Interact.

[12] Lo Presti L, Lanver D, Schweizer G, Tanaka S, Liang L, Tollot M, Zuccaro A, Reissmann S, Kahmann R (2015). Fungal effectors and plant susceptibility. Annu Rev Plant Biol.

[13] Białas A, Zess EK, De La Concepcion JC, Franceschetti M, Pennington HG, Yoshida K (2018). Lessons in effector and NLR biology of plant-microbe systems. Mol Plant-Microbe Interact.

[14] Mcgowan J, Fitzpatrick DA (2017). Genomic, Network, and Phylogenetic Analysis of the Oomycete Effector Arsenal.

[15] Fouché S, Mence Plissonneau C, Croll D (2018). The birth and death of effectors in rapidly evolving filamentous pathogen genomes.

[16] Hartmann FE, Sánchez-Vallet A, McDonald BA, Croll D (2017). A fungal wheat pathogen evolved host specialization by extensive chromosomal rearrangements. ISME J.

[17] Zhong Z, Marcel TC, Hartmann FE, Ma X, Plissonneau C, Zala M, Ducasse A, Confais J, Compain J, Lapalu N, Amselem J, McDonald BA, Croll D, Palma-Guerrero J (2017). A small secreted protein in Zymoseptoria tritici is responsible for avirulence on wheat cultivars carrying the Stb6 resistance gene. New Phytol.

[18] Gohari AM, Ware SB, Wittenberg AHJ, Mehrabi R, Ben M’BS, ECP V (2015). Effector discovery in the fungal wheat pathogen Zymoseptoria tritici. Mol Plant Pathol.

[19] Meile L, Croll D, Brunner PC, Plissonneau C, Hartmann FE, McDonald BA (2018). A fungal avirulence factor encoded in a highly plastic genomic region triggers partial resistance to septoria tritici blotch. New Phytol.

[20] Stewart E L., Croll D, Lendenmann MH, Sanchez-Vallet a, Hartmann FE, Palma-Guerrero J, et al. quantitative trait locus mapping reveals complex genetic architecture of quantitative virulence in the wheat pathogen *Zymoseptoria tritici*. Mol Plant Pathol 2018;19:201–216. doi:10.1111/mpp.12515, 1. 10.1111/mpp.12515 PMC6638037 27868326

[21] Kema GHJ, Mirzadi Gohari A, Aouini L, Gibriel HAY, Ware SB, van den Bosch F, Manning-Smith R, Alonso-Chavez V, Helps J, Ben M’Barek S, Mehrabi R, Diaz-Trujillo C, Zamani E, Schouten HJ, van der Lee TAJ, Waalwijk C, de Waard MA, de Wit PJGM, Verstappen ECP, Thomma BPHJ, Meijer HJG, Seidl MF (2018). Stress and sexual reproduction affect the dynamics of the wheat pathogen effector AvrStb6 and strobilurin resistance. Nat Genet.

[22] Plissonneau C, Hartmann FE, Croll D (2018). Pangenome analyses of the wheat pathogen Zymoseptoria tritici reveal the structural basis of a highly plastic eukaryotic genome. BMC Biol.

[23] Plissonneau C, Stürchler A, Croll D, Taylor JW. The Evolution of Orphan Regions in Genomes of a Fungal Pathogen of Wheat. 2016;7(5). 10.1128/mBio.01231-16. 10.1128/mBio.01231-16 PMC5082898 27795389

[24] Badet T, Oggenfuss U, Abraham L, McDonald BA, Croll D (2020). A 19-isolate reference-quality global pangenome for the fungal wheat pathogen Zymoseptoria tritici. BMC Biol.

[25] Badet T, Croll D (2020). The rise and fall of genes: origins and functions of plant pathogen pangenomes. Curr Opin Plant Biol.

[26] Dong S, Raffaele S, Kamoun S (2015). The two-speed genomes of filamentous pathogens: waltz with plants. Curr Opin Genet Dev.

[27] Asai S, Furzer OJ, Cevik V, Kim DS, Ishaque N, Goritschnig S, Staskawicz BJ, Shirasu K, Jones JDG (2018). A downy mildew effector evades recognition by polymorphism of expression and subcellular localization. Nat Commun.

[28] Dong S, Qutob D, Tedman-Jones J, Kuflu K, Wang Y, Tyler BM, Gijzen M (2009). The Phytophthora sojae Avirulence locus Avr3c encodes a multi-copy RXLR effector with sequence polymorphisms among pathogen strains. PLoS One.

[29] Cowger C, Brown JKM (2019). Durability of quantitative resistance in crops: greater than we know?. Annu Rev Phytopathol.

[30] Cowger C, Hoffer ME, Mundt CC (2000). Specific adaptation by Mycosphaerella graminicola to a resistant wheat cultivar. Plant Pathol.

[31] Longya A, Chaipanya C, Franceschetti M, Maidment JHR, Banfield MJ, Jantasuriyarat C (2019). Gene duplication and mutation in the emergence of a novel aggressive allele of the *AVR-Pik* effector in the Rice blast fungus. Mol Plant-Microbe Interact.

[32] Islam MT, Croll D, Gladieux P, Soanes DM, Persoons A, Bhattacharjee P, Hossain MS, Gupta DR, Rahman MM, Mahboob MG, Cook N, Salam MU, Surovy MZ, Sancho VB, Maciel JLN, NhaniJúnior A, Castroagudín VL, Reges JTA, Ceresini PC, Ravel S, Kellner R, Fournier E, Tharreau D, Lebrun MH, McDonald BA, Stitt T, Swan D, Talbot NJ, Saunders DGO, Win J, Kamoun S (2016). Emergence of wheat blast in Bangladesh was caused by a south American lineage of Magnaporthe oryzae. BMC Biol.

[33] Frantzeskakis L, Di Pietro A, Rep M, Schirawski J, Wu C, Panstruga R (2020). Rapid evolution in plant–microbe interactions – a molecular genomics perspective. New Phytol.

[34] Rouxel T, Balesdent M-H (2017). Life, death and rebirth of avirulence effectors in a fungal pathogen of Brassica crops, *Leptosphaeria maculans*. New Phytol.

[35] Ma L-J, van der Does HC, Borkovich KA, Coleman JJ, Daboussi M-J, Di Pietro A (2010). Comparative genomics reveals mobile pathogenicity chromosomes in Fusarium. Nature..

[36] Manning VA, Pandelova I, Dhillon B, Wilhelm LJ, Goodwin SB, Berlin AM, Figueroa M, Freitag M, Hane JK, Henrissat B, Holman WH, Kodira CD, Martin J, Oliver RP, Robbertse B, Schackwitz W, Schwartz DC, Spatafora JW, Turgeon BG, Yandava C, Young S, Zhou S, Zeng Q, Grigoriev IV, Ma LJ, Ciuffetti LM (2013). Comparative genomics of a plant-pathogenic fungus, Pyrenophora tritici-repentis, reveals Transduplication and the impact of repeat elements on pathogenicity and population divergence. G3 genes, genomes. Genet..

[37] Croll D, McDonald BA (2012). The accessory genome as a cradle for adaptive evolution in pathogens. PLoS Pathog.

[38] Wang Q, Jiang C, Wang C, Chen C, Xu J-R, Liu H. Characterization of the two-speed subgenomes of Fusarium graminearum reveals the fast-speed subgenome specialized for adaption and infection. Front Plant Sci. 2017;8. 10.3389/fpls.2017.00140. 10.3389/fpls.2017.00140 PMC5306128 28261228

[39] Torres DE, Oggenfuss U, Croll D, Seidl MF (2020). Genome evolution in fungal plant pathogens: looking beyond the two-speed genome model. Fungal Biol Rev.

[40] Xue M, Yang J, Li Z, Hu S, Yao N, Dean RA, Zhao W, Shen M, Zhang H, Li C, Liu L, Cao L, Xu X, Xing Y, Hsiang T, Zhang Z, Xu JR, Peng YL (2012). Comparative analysis of the genomes of two field isolates of the Rice blast fungus Magnaporthe oryzae. PLoS Genet.

[41] Yoshida K, Saunders DGO, Mitsuoka C, Natsume S, Kosugi S, Saitoh H, Inoue Y, Chuma I, Tosa Y, Cano LM, Kamoun S, Terauchi R (2016). Host specialization of the blast fungus Magnaporthe oryzae is associated with dynamic gain and loss of genes linked to transposable elements. BMC Genomics.

[42] Chuma I, Isobe C, Hotta Y, Ibaragi K, Futamata N, Kusaba M, Yoshida K, Terauchi R, Fujita Y, Nakayashiki H, Valent B, Tosa Y (2011). Multiple translocation of the AVR-Pita effector gene among chromosomes of the Rice blast fungus Magnaporthe oryzae and related species. PLoS Pathog.

[43] Wu J, Kou Y, Bao J, Li Y, Tang M, Zhu X, Ponaya A, Xiao G, Li J, Li C, Song MY, Cumagun CJR, Deng Q, Lu G, Jeon JS, Naqvi NI, Zhou B (2015). Comparative genomics identifies the *Magnaporthe oryzae* avirulence effector *AvrPi9* that triggers *Pi9* -mediated blast resistance in rice. New Phytol.

[44] Fouché S, Badet T, Oggenfuss U, Plissonneau C, Francisco CS, Croll D (2020). Stress-driven transposable element De-repression dynamics and virulence evolution in a fungal pathogen. Mol Biol Evol.

[45] Sánchez-Vallet A, Fouché S, Fudal I, Hartmann FE, Soyer JL, Tellier A (2018). The genome biology of effector gene evolution in filamentous plant pathogens. Annu Rev Phytopathol.

[46] Gladyshev E (2017). Repeat-induced point mutation and other genome defense mechanisms in Fungi. The fungal kingdom.

[47] Gardiner DM, Rusu A, Barrett L, Hunter GC, Kazan K (2020). Can natural gene drives be part of future fungal pathogen control strategies in plants?. New Phytol.

[48] Wang L, Sun Y, Sun X, Yu L, Xue L, He Z, Huang J, Tian D, Hurst LD, Yang S (2020). Repeat-induced point mutation in Neurospora crassa causes the highest known mutation rate and mutational burden of any cellular life. Genome Biol.

[49] Van de Wouw AP, Cozijnsen AJ, Hane JK, Brunner PC, McDonald BA, Oliver RP (2010). Evolution of linked Avirulence effectors in Leptosphaeria maculans is affected by genomic environment and exposure to resistance genes in host plants. PLoS Pathog.

[50] Fones H, Gurr S (2015). The impact of Septoria tritici blotch disease on wheat: an EU perspective. Fungal Genet Biol.

[51] Jørgensen LN, Hovmøller MS, Hansen JG, Lassen P, Clark B, Bayles R, Rodemann B, Flath K, Jahn M, Goral T, Jerzy Czembor J, Cheyron P, Maumene C, de Pope C, Ban R, Nielsen GC, Berg G (2014). IPM strategies and their dilemmas including an introduction to www.eurowheat.org. J Integr Agric.

[52] Hartmann FE, Croll D (2017). Distinct trajectories of massive recent gene gains and losses in populations of a microbial eukaryotic pathogen. Mol Biol Evol.

[53] Krishnan P, Ma X, McDonald BA, Brunner PC (2018). Widespread signatures of selection for secreted peptidases in a fungal plant pathogen. BMC Evol Biol.

[54] Singh NK, Chanclud E, Croll D. Population-level deep sequencing reveals the interplay of clonal and sexual reproduction in the fungal wheat pathogen Zymoseptoria tritici. bioRxiv. 2020:2020.07.07.191510. 10.1101/2020.07.07.191510. 10.1099/mgen.0.000678 PMC8627204 34617882

[55] Dutta A, Hartmann FE, Francisco CS, McDonald BA, Croll D. Mapping the adaptive landscape of a major agricultural pathogen reveals evolutionary constraints across heterogeneous environments. ISME J. 2021:1–18. 10.1038/s41396-020-00859-w. 10.1038/s41396-020-00859-w PMC8115182 33452474

[56] Meile L, Peter J, Puccetti G, Alassimone J, McDonald BA, Sánchez-Vallet A (2020). Chromatin dynamics contribute to the spatiotemporal expression pattern of virulence genes in a fungal plant pathogen. MBio..

[57] Dutta A, Croll D, McDonald BA, Barrett LG. Maintenance of variation in virulence and reproduction in populations of an agricultural plant pathogen. Evol Appl. 2020:eva.13117. 10.1111/eva.13117. 10.1111/eva.13117 PMC7896723 33664780

[58] Brown JKM, Chartrain L, Lasserre-Zuber P, Saintenac C (2015). Genetics of resistance to Zymoseptoria tritici and applications to wheat breeding. Fungal Genet Biol.

[59] Karisto P, Hund A, Yu K, Anderegg J, Walter A, Mascher F, et al. Ranking quantitative resistance to Septoria tritici blotch in elite wheat cultivars using automated image analysis. bioRxiv. 2017. 10.1101/129353. 10.1094/PHYTO-04-17-0163-R 29210601

[60] Courvoisier N, Häner LL, Bertossa M, Thévoz E, Anders M, Stoll P (2016). céréales-variétés 2.21 Blé d’automne Juin 2016.

[61] Mikaberidze A, McDonald BA (2020). A tradeoff between tolerance and resistance to a major fungal pathogen in elite wheat cultivars. New Phytol.

[62] Zhao S, Ye Z, Stanton R (2020). Misuse of RPKM or TPM normalization when comparing across samples and sequencing protocols. RNA..

[63] Palma-Guerrero J, Ma X, Torriani SFF, Zala M, Francisco CS, Hartmann FE, Croll D, McDonald BA (2017). Comparative Transcriptome analyses in Zymoseptoria tritici reveal significant differences in gene expression among strains during plant infection. Mol Plant-Microbe Interact.

[64] Torriani SFF, Stukenbrock EH, Brunner PC, McDonald BA, Croll D (2011). Evidence for extensive recent intron transposition in closely related Fungi. Curr Biol.

[65] Mohd-Assaad N, McDonald BA, Croll D (2019). The emergence of the multi-species NIP1 effector in *Rhynchosporium* was accompanied by high rates of gene duplications and losses. Environ Microbiol.

[66] Stauber L, Prospero S, Croll D. Comparative Genomics Analyses of Lifestyle Transitions at the Origin of an Invasive Fungal Pathogen in the Genus Cryphonectria. mSphere. 2020;5. 10.1128/MSPHERE.00737-20. 10.1128/mSphere.00737-20 PMC7565894 33055257

[67] Altschul SF, Gish W, Miller W, Myers EW, Lipman DJ (1990). Basic local alignment search tool. J Mol Biol.

[68] Dickman MB, de Figueiredo P (2013). Death be not proud—cell death control in plant fungal interactions. PLoS Pathog.

[69] Coll NS, Epple P, Dangl JL (2011). Programmed cell death in the plant immune system. Cell Death Differ.

[70] Beckerson WC, de la Vega RCR, Hartmann FE, Duhamel M, Giraud T, Perlin MH. Cause and effectors: whole-genome comparisons reveal shared but rapidly evolving effector sets among host-specific plant-castrating Fungi. MBio. 2019;10(6). 10.1128/MBIO.02391-19. 10.1128/mBio.02391-19 PMC6831777 31690676

[71] Zeilinger S, Gupta VK, Dahms TES, Silva RN, Singh HB, Upadhyay RS, Gomes EV, Tsui CKM, Nayak S C (2016). Friends or foes? Emerging insights from fungal interactions with plants. FEMS Microbiol Rev.

[72] Karisto P, Hund A, Yu K, Anderegg J, Walter A, Mascher F, McDonald BA, Mikaberidze A (2018). Ranking quantitative resistance to septoria tritici blotch in elite wheat cultivars using automated image analysis. Phytopathology..

[73] Muszewska A, Stepniewska-Dziubinska MM, Steczkiewicz K, Pawlowska J, Dziedzic A, Ginalski K (2017). Fungal lifestyle reflected in serine protease repertoire. Sci Rep.

[74] Langner T, Göhre V (2016). Fungal chitinases: function, regulation, and potential roles in plant/pathogen interactions. Curr Genet.

[75] Jashni MK, Dols IHM, Iida Y, Boeren S, Beenen HG, Mehrabi R, Collemare J, de Wit PJGM (2015). Synergistic action of a Metalloprotease and a serine protease from *Fusarium oxysporum* f. sp. *lycopersici* cleaves chitin-binding tomato Chitinases, reduces their antifungal activity, and enhances fungal virulence. Mol Plant-Microbe Interact.

[76] Palma-Guerrero J, Torriani SFF, Zala M, Carter D, Courbot M, Rudd JJ, McDonald BA, Croll D (2016). Comparative transcriptomic analyses of Zymoseptoria tritici strains show complex lifestyle transitions and intraspecific variability in transcription profiles. Mol Plant Pathol.

[77] Krishnan P, Meile L, Plissonneau C, Ma X, Hartmann FE, Croll D, McDonald BA, Sánchez-Vallet A (2018). Transposable element insertions shape gene regulation and melanin production in a fungal pathogen of wheat. BMC Biol.

[78] Omrane S, Audéon C, Ignace A, Duplaix C, Aouini L, Kema G, et al. Plasticity of the MFS1 Promoter Leads to Multidrug Resistance in the Wheat Pathogen Zymoseptoria tritici. mSphere. 2017;2. 10.1128/MSPHERE.00393-17. 10.1128/mSphere.00393-17 PMC5656749 29085913

[79] Rebollo R, Romanish MT, Mager DL (2012). Transposable elements: an abundant and natural source of regulatory sequences for host genes. Annu Rev Genet.

[80] Cowley M, Oakey RJ (2013). Transposable elements re-wire and fine-tune the Transcriptome. PLoS Genet.

[81] Bennetzen JL, Wang H (2014). The contributions of transposable elements to the structure, function, and evolution of plant genomes. Annu Rev Plant Biol.

[82] Santana MF, Silva JC, Batista AD, Ribeiro LE, da Silva GF, de Araújo EF (2012). Abundance, distribution and potential impact of transposable elements in the genome of Mycosphaerella fijiensis. BMC Genomics.

[83] Santana MF, Silva JC, Mizubuti ES, Araújo EF, Condon BJ, Turgeon B (2014). Characterization and potential evolutionary impact of transposable elements in the genome of Cochliobolus heterostrophus. BMC Genomics.

[84] Sperschneider J, Gardiner DM, Thatcher LF, Lyons R, Singh KB, Manners JM, Taylor JM (2015). Genome-wide analysis in three Fusarium pathogens identifies rapidly evolving chromosomes and genes associated with pathogenicity. Genome Biol Evol.

[85] Faino L, Seidl MF, Shi-Kunne X, Pauper M, van den Berg GCM, Wittenberg AHJ, Thomma BPHJ (2016). Transposons passively and actively contribute to evolution of the two-speed genome of a fungal pathogen. Genome Res.

[86] Levy L, Courvoisier N, Rechsteiner S, Herrera J, Brabant C, Hund A (2017). Winterweizen: Bilanz aus 15 Jahren Sortenprüfung unter extensiven Anbaubedingungen. Agrar Schweiz.

[87] Stewart EL, Hagerty CH, Mikaberidze A, Mundt C, Zhong Z, McDonald BA (2016). An improved method for measuring quantitative resistance to the wheat pathogen Zymoseptoria tritici using high throughput automated image analysis. Phytopathology..

[88] Oggenfuss U, Badet T, Wicker T, Hartmann FE, Singh NK, Abraham LN, et al. A population-level invasion by transposable elements in a fungal pathogen. bioRxiv. 2020:2020.02.11.944652. 10.1101/2020.02.11.944652. 10.7554/eLife.69249 PMC8445621 34528512

[89] Vogel HJ (1956). A convenient growth medium for Neurospora crassa. Microb Genet Bull.

[90] Metzenberg RL (2003). Vogel’s medium N salts: avoiding the need for ammonium nitrate. Fungal Genet Rep.

[91] Bolger AM, Lohse M, Usadel B (2014). Trimmomatic: a flexible trimmer for Illumina sequence data. Bioinformatics..

[92] Goodwin SB, M’Barek SB, Dhillon B, AHJ W, Crane CF, Hane JK (2011). Finished genome of the fungal wheat pathogen Mycosphaerella graminicola reveals dispensome structure, chromosome plasticity, and stealth pathogenesis. PLoS Genet.

[93] Langmead B, Salzberg SL (2012). Fast gapped-read alignment with bowtie 2. Nat Methods.

[94] McKenna A, Hanna M, Banks E, Sivachenko A, Cibulskis K, Kernytsky A, Garimella K, Altshuler D, Gabriel S, Daly M, DePristo MA (2010). The genome analysis toolkit: a MapReduce framework for analyzing next-generation DNA sequencing data. Genome Res.

[95] Danecek P, Auton A, Abecasis G, Albers CA, Banks E, DePristo MA (2011). The variant call format and VCFtools. Bioinformatics..

[96] Kim D, Paggi JM, Park C, Bennett C, Salzberg SL (2019). Graph-based genome alignment and genotyping with HISAT2 and HISAT-genotype. Nat Biotechnol.

[97] Bergelson J, Roux F (2010). Towards identifying genes underlying ecologically relevant traits in Arabidopsis thaliana. Nat Rev Genet.

[98] Korte A, Farlow A (2013). The advantages and limitations of trait analysis with GWAS: a review. Plant Methods.

[99] Knaus BJ, Grünwald NJ. vcfr: a package to manipulate and visualize variant call format data in R. In: Molecular Ecology Resources: John Wiley & Sons, Ltd; 2017. p. 44–53. 10.1111/1755-0998.12549. 10.1111/1755-0998.12549 27401132

[100] Jombart T, Ahmed I (2011). Adegenet 1.3-1: new tools for the analysis of genome-wide SNP data. Bioinformatics..

[101] Dray S, Dufour AB (2007). The ade4 package: Implementing the duality diagram for ecologists. J Stat Softw.

[102] Wickham H (2016). Ggplot2 : elegant graphics for data analysis.

[103] Huson DH (1998). SplitsTree: analyzing and visualizing evolutionary data. Bioinformatics..

[104] Lischer HEL, Excoffier L (2012). PGDSpider: an automated data conversion tool for connecting population genetics and genomics programs. Bioinformatics..

[105] Paradis E, Schliep K (2019). Ape 5.0: an environment for modern phylogenetics and evolutionary analyses in R. Bioinformatics..

[106] Yang J, Lee SH, Goddard ME, Visscher PM (2011). GCTA: a tool for genome-wide complex trait analysis. Am J Hum Genet.

[107] Bradbury PJ, Zhang Z, Kroon DE, Casstevens TM, Ramdoss Y, Buckler ES (2007). TASSEL: software for association mapping of complex traits in diverse samples. Bioinformatics..

[108] Schloerke B, Briatte F, Joseph b, elbamos CJ (2021). ggobi/ggally: v2.1.1.

[109] Turner SD. qqman: an R package for visualizing GWAS results using Q-Q and manhattan plots. bioRxiv. 2014:005165. 10.1101/005165.

[110] Quinlan AR, Hall IM (2010). BEDTools: a flexible suite of utilities for comparing genomic features. Bioinformatics..

[111] Shin JH, Blay S, McNeney B, Graham J. LDheatmap: An R function for graphical display of pairwise linkage disequilibria between single nucleotide polymorphisms. J Stat Softw. 2006;16:1–9. doi:10.18637/jss.v016.c03.

[112] Guy L, Roat Kultima J, Andersson SGE (2010). genoPlotR: comparative gene and genome visualization in R. Bioinformatics..

[113] van Wyk S, Harrison CH, Wingfield BD, De Vos L, van der Merwe NA, Steenkamp ET (2019). The RIPper, a web-based tool for genome-wide quantification of repeat-induced point (RIP) mutations. PeerJ..

[114] Bankevich A, Nurk S, Antipov D, Gurevich AA, Dvorkin M, Kulikov AS, Lesin VM, Nikolenko SI, Pham S, Prjibelski AD, Pyshkin AV, Sirotkin AV, Vyahhi N, Tesler G, Alekseyev MA, Pevzner PA (2012). SPAdes: a new genome assembly algorithm and its applications to single-cell sequencing. J Comput Biol.

[115] Bao W, Kojima KK, Kohany O (2015). Repbase update, a database of repetitive elements in eukaryotic genomes. Mob DNA.

[116] Wicker T, Sabot F, Hua-Van A, Bennetzen JL, Capy P, Chalhoub B (2007). A unified classification system for eukaryotic transposable elements. Nat Rev Genet.

[117] Smit A, Hubley R (2015). RepeatModeler Open-1.0.

