# Supplementary material for: Rapid sequence evolution driven by transposable elements at a virulence locus in a fungal wheat pathogen
Source: BMC Genomics. 2021 May 27;22:393. doi: 10.1186/s12864-021-07691-2 (PMC8157644; doi:10.1186/s12864-021-07691-2)
Supplement: Supplementary file 1 — Additional file 1: Supplementary Fig. 1: The first two principal components (PC) of 788′313 genome-wide SNPs in 114/120 isolates. Isolates are color-coded by the cultivar of the origin. The six excluded isolates grouped into two clone groups of three isolates each and were collected from cultivar CH Combin. Supplementary Fig. 2: Phenotypic trait values. Red and blue lines represent the mean and median, respectively. A) The percentage of leaf area covered by lesion (PLACL). B) pycnidia count. C) Tolerance expressed as the pycnidia count divided by PLACL. D) ρleaf is the pycnidia count per cm2 of leaf area and E) ρlesion is defined as the total number of pycnidia divided by per cm2 lesion area. Supplementary Fig. 3: Manhattan and QQ-plot representing of the genome-wide association mapping analyses using mixed linear models based on a kinship matrix. The blue line corresponds to the Bonferroni threshold (alpha = 0.05) and the red line corresponds to the 5% FDR. A-B) Percentage of leaf area covered by lesions (PLACL), C-D) tolerance E-F) ρlesion. Supplementary Fig. 4: Manhattan and QQ-plot representing of the genome-wide association mapping analyses. The GWAS was performed using mixed linear models including a kinship matrix and the first two principal components as random factors. The blue line corresponds to the Bonferroni threshold (alpha = 0.05) and the red line corresponds to the 5% FDR. A-B) Percentage of leaf area covered by lesions (PLACL), C-D) Pycnidia count, E-F) tolerance G-H) ρleaf and I-J) ρlesion. Supplementary Fig. 5: Gene expression in reads per kilobase of transcript, per million mapped reads (RPKM) for genes closest to the top-associated SNP. A) Putative effector gene (Zt09_1_01590) and B) serine-type endopeptidase gene (Zt09_1_01591). Supplementary Fig. 6: The Large RIP Affected Regions (LRARs) composite index was calculated using The RIPer tool (van Wyk et al., 2019) and shown in black. The region flanked by the genes Zt09_1_01590 and Zt09_1_01591 i [file 12864_2021_7691_MOESM1_ESM.pdf]

SUPPLEMENTARY INFORMATION

Rapid sequence evolution driven by transposable elements at a virulence locus in a fungal wheat pathogen

Nikhil Kumar Singh[1], Thomas Badet[1], Leen Abraham[1], Daniel Croll[1,*]

[1] Laboratory of Evolutionary Genetics, Institute of Biology, University of Neuchâtel, 2000 Neuchâtel, Switzerland

[*] Author for correspondence: daniel.croll@unine.ch

Supplementary Figures

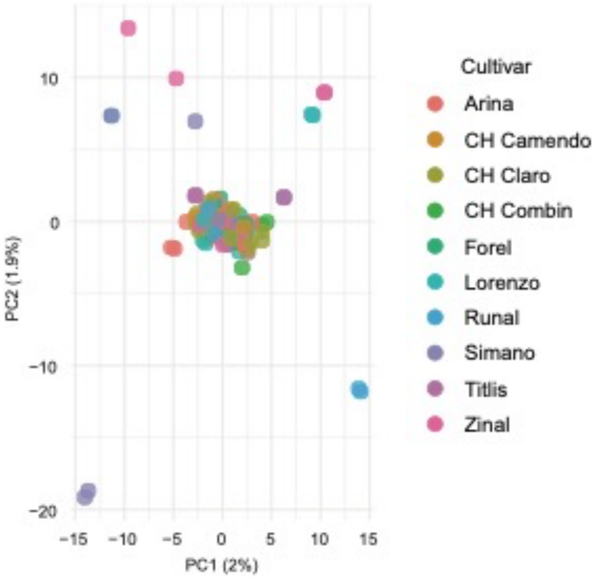

Supplementary Figure 1: The first two principal components (PC) of 788’313 genome-wide SNPs in 114/120 isolates. Isolates are color-coded by the cultivar of the origin. The six excluded isolates grouped into two clone groups of three isolates each and were collected from cultivar CH Combin.

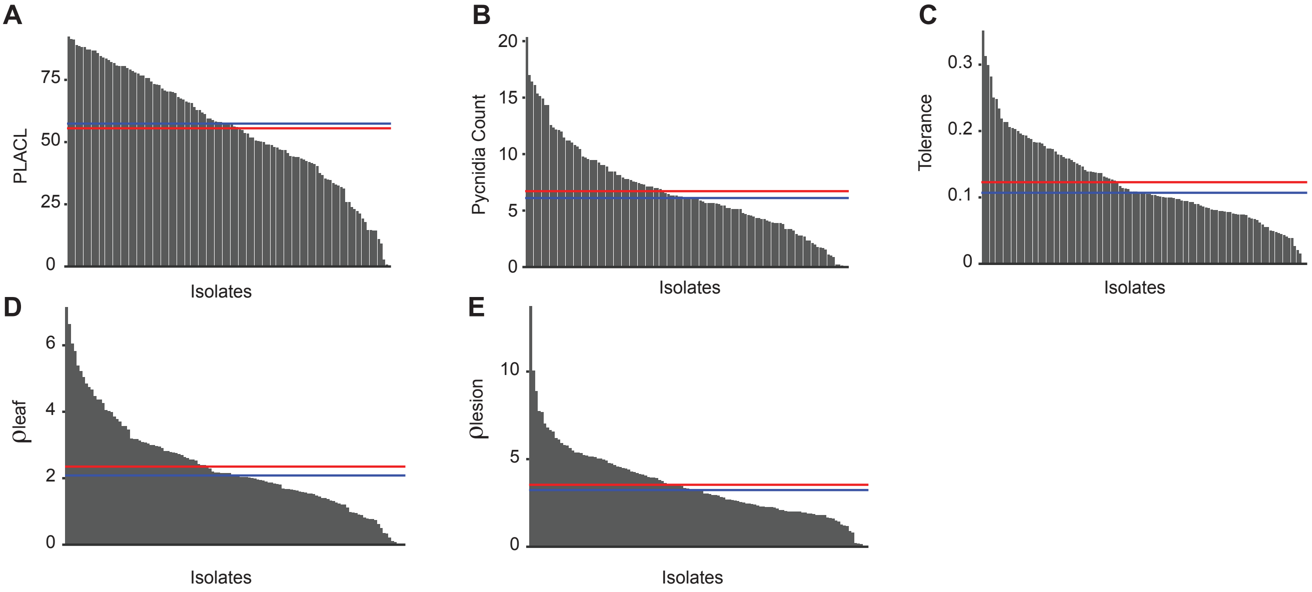

Supplementary Figure 2: Phenotypic trait values. Red and blue lines represent the mean and median, respectively. A) The percentage of leaf area covered by lesion (PLACL). B) pycnidia count. C) Tolerance expressed as the pycnidia count divided by PLACL. D) ρ_{leaf} is the pycnidia count per cm^2 of leaf area and E) ρ_{lesion} is defined as the total number of pycnidia divided by per cm^2 lesion area.

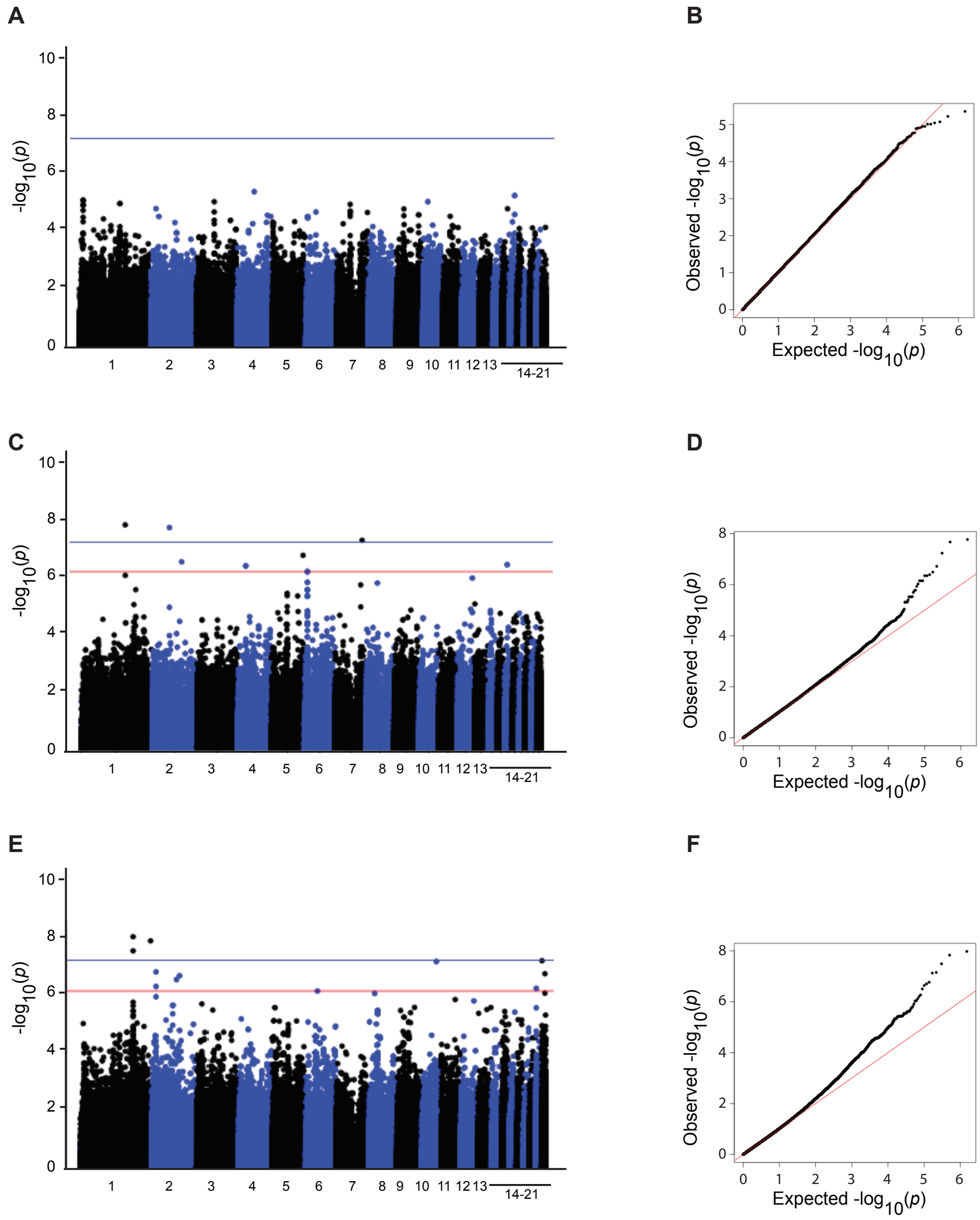

Supplementary Figure 3: Manhattan and QQ-plot representing of the genome-wide association mapping analyses using mixed linear models based on a kinship matrix. The blue line corresponds to the Bonferroni threshold (alpha = 0.05) and the red line corresponds to the 5% FDR. A-B) Percentage of leaf area covered by lesions (PLACL), C-D) tolerance E-F) ρ_{lesion}

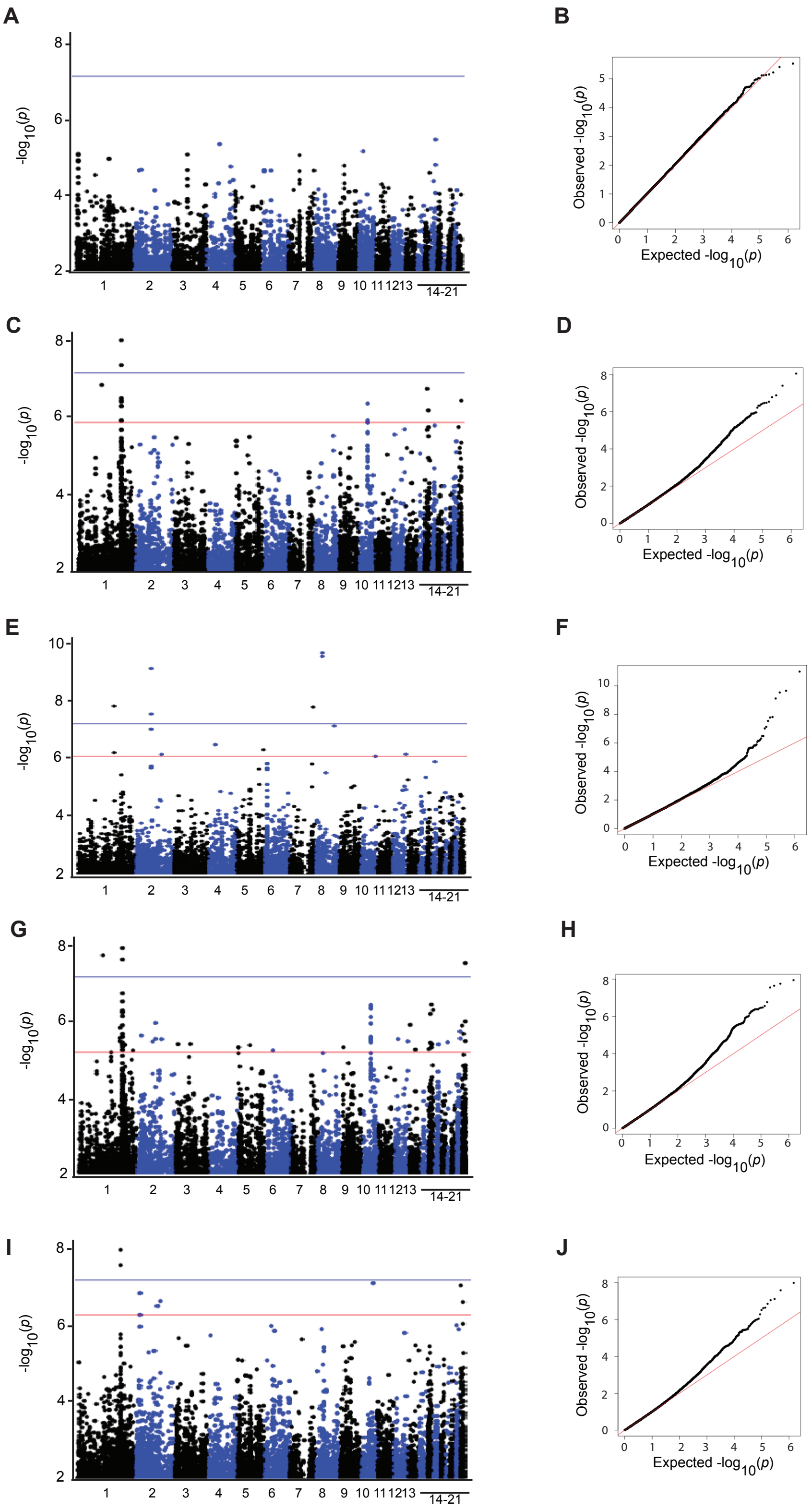

Supplementary Figure 4: Manhattan and QQ-plot representing of the genome-wide association mapping analyses. The GWAS was performed using mixed linear models including a kinship matrix and the first two principal components as random factors. The blue line corresponds to the Bonferroni threshold (alpha = 0.05) and the red line corresponds to the 5% FDR. A-B) Percentage of leaf area covered by lesions (PLACL), C-D) Pycnidia count, E-F) tolerance G-H) ρ_{leaf} and I-J) ρ_{lesion}

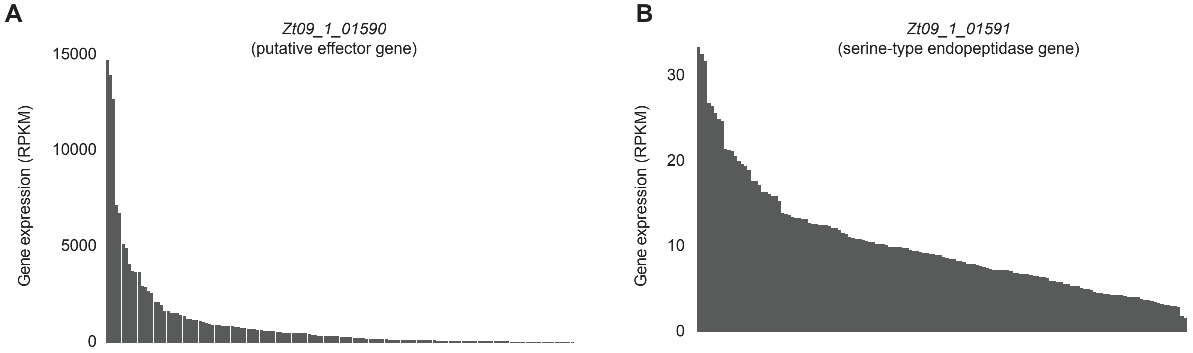

Supplementary Figure 5: Gene expression in reads per kilobase of transcript, per million mapped reads (RPKM) for genes closest to the top-associated SNP. A) Putative effector gene (*Zt09_1_01590*) and B) serine-type endopeptidase gene (Zt09_1_01591).

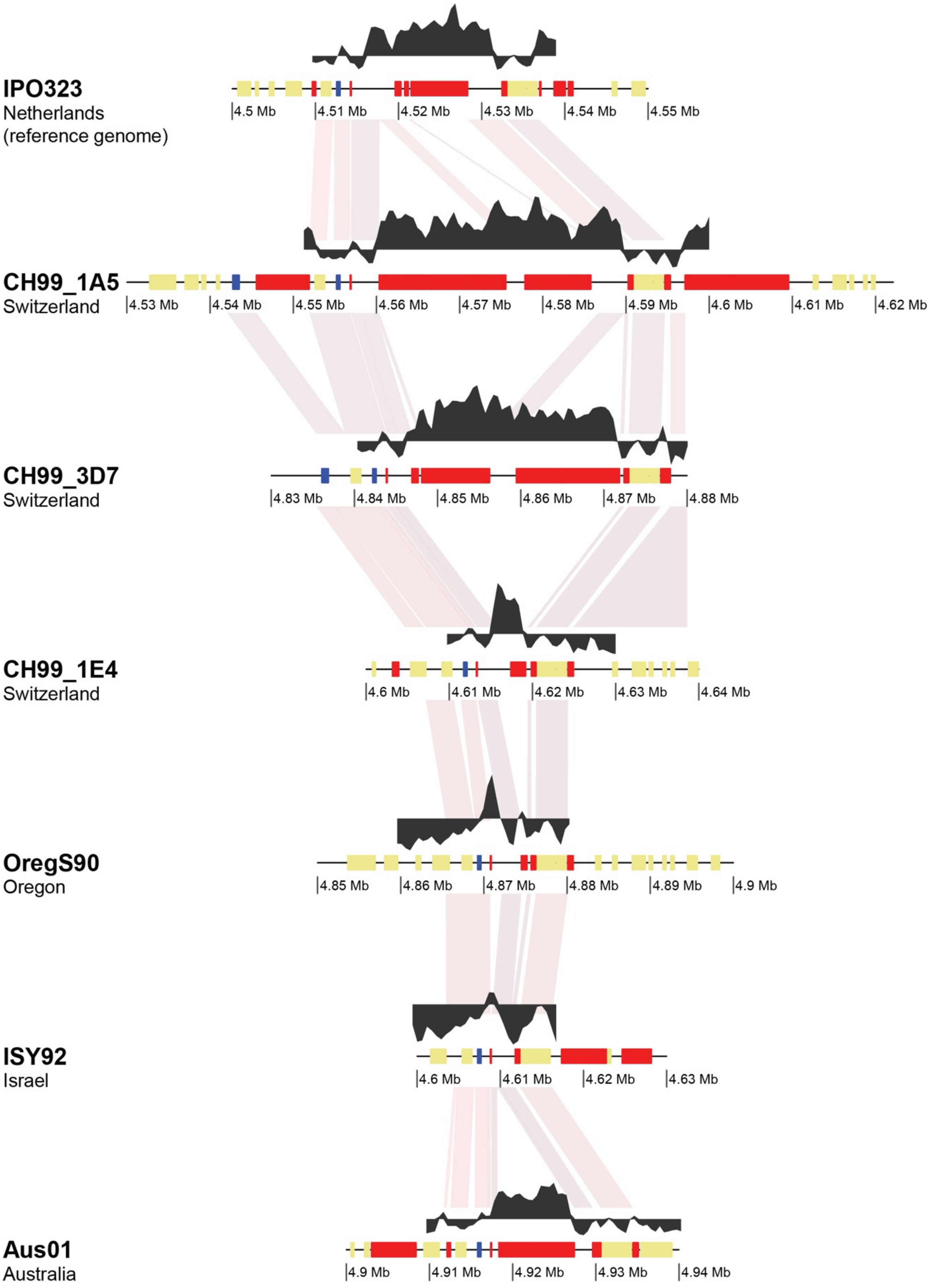

Supplementary Figure 6 : The Large RIP Affected Regions (LRARs) composite index was calculated using The RIPer tool (van Wyk et al., 2019) and shown in black. The region flanked by the genes *Zt09_1_01590* and *Zt09_1_01591* is shown in complete genome assemblies of seven isolates from global population.

Supplementary Tables

(see separate Excel file)

Supplementary Table S1: Phenotypic trait values used for GWAS. The percentage of leaf area covered by lesion (PLACL); ρ_{leaf} is the pycnidia count per cm^2 of leaf area; ρ_{lesion} is defined as the total number of pycnidia divided by per cm^2 lesion area. Tolerance is expressed as the pycnidia count divided by PLACL

Supplementary Table S2: Groups of clonal genotypes identified in the GWAS population with information about the collection time point and cultivar of origin. The clonal genotype columns provides a unique identifier. See Singh *et al.* (2020) for more detailed analyses.

Supplementary Table S3: List of significantly associated SNPs above 5% FDR for pycnidia counts .

Supplementary Table S4: Number of isolates from populations on different continents analyzed for transposable element content. Total assembled genomes and total of isolates per population where a scaffold was retrieved containing both genes *Zt09_1_01590* and *Zt09_1_01591*.
